# N-of-one differential gene expression without control samples using a deep generative model

**DOI:** 10.1186/s13059-023-03104-7

**Published:** 2023-11-16

**Authors:** Iñigo Prada-Luengo, Viktoria Schuster, Yuhu Liang, Thilde Terkelsen, Valentina Sora, Anders Krogh

**Affiliations:** 1 Department of Computer Science, University of Copenhagen, Copenhagen, Denmark; 2 Center for Health Data Science, University of Copenhagen, Copenhagen, Denmark

**Keywords:** Deep generative models, Deep learning, Differential expression analysis, DEG, DEseq2, Transcriptomics

## Abstract

Differential analysis of bulk RNA-seq data often suffers from lack of good controls. Here, we present a generative model that replaces controls, trained solely on healthy tissues. The unsupervised model learns a low-dimensional representation and can identify the closest normal representation for a given disease sample. This enables control-free, single-sample differential expression analysis. In breast cancer, we demonstrate how our approach selects marker genes and outperforms a state-of-the-art method. Furthermore, significant genes identified by the model are enriched in driver genes across cancers. Our results show that the in silico closest normal provides a more favorable comparison than control samples.

**Supplementary Information:**

The online version contains supplementary material available at 10.1186/s13059-023-03104-7.

## Background

Cellular function varies with cell type and environment and differences in cell function can largely be characterized by gene expression profiles [1, 2]. The analysis of gene expression data has become a standard for studying differences in cells and tissues, partly driven by advances in next-generation RNA sequencing (RNA-Seq) technologies. By identifying differentially expressed genes (DEGs), we can pinpoint genes involved in the onset or progression of disease, which could represent biomarkers or potential drug targets in personalized treatment [3, 4].

Despite the great potential of differential expression analysis, the methods employed for this type of analysis yield results which are often not reproducible and may return thousands of significant DEGs [5], making a clinical interpretation challenging. This drawback is largely due to the lack of good controls, a common problem in the study of diseases. In cancer studies, controls are most often tissue samples from healthy individuals (potentially matched on available clinical parameters) or normal adjacent tissues (NATs) from the cancer patients. The benefit of the latter is a reduction of person-specific biological variance. However, NATs from cancer patients have been shown to display some of the same characteristics as the tumor tissues [6], implying that these samples are not truly normal. Inversely, normal samples from other individuals will display genetic heterogeneity and are thus unsuitable for direct comparison with classical methods, especially at low sample numbers [7, 8]. Lastly, a general problem concerning bulk sequencing data is that samples differ in cell type composition. This problem may in part be alleviated by using weighted averages of the closest normal samples [9, 10].

Most often differential expression analysis relies on negative binomial generalized linear models, which can account for the over-dispersion associated with count data [11]. Neural networks and machine learning in general have been increasingly applied to transcriptomics in the last two decades. Applications range from quality control using simple regression and mixture models [12] over identifying DEGs and biomarkers using random forests [13] or convolutional neural networks [14] to digital pathology [15] using multi-layer perceptrons. Other approaches have been suggested to learn biologically meaningful representations from gene expression data. Way and Greene [16] present a variational autoencoder [17] trained on cancer transcriptomes with the potential to predict therapeutic responses. Another generative neural network, SOPHIE [18], identifies cancer-specific genes from a collection of normal and cancer datasets. So far, available methods seem to be limited to requiring paired or manually curated controls or training on particular datasets, including cancer samples.

In this work, we present a gene expression model of normal tissue that replaces control samples and enables differential expression analysis in cancer using only a single sample. The model is based on the Deep Generative Decoder (DGD) [19, 20], a generative neural network that learns a probabilistic low-dimensional representation of the data. Our model is trained on the Genotype-Tissue Expression (GTEx) dataset [2] containing around 20,000 bulk samples from 31 different human tissues and 948 individuals. Briefly, the model learns parameters with two aims. Firstly, the neural network parameters in the decoder are learned to best describe all data in a low-dimensional space (the “representation”). Secondly, the model learns the most probable representation for each sample in the low-dimensional space and returns a negative binomial distribution over count values for every gene. For samples derived from non-normal tissue, such as cancer samples, we can infer the nearest representation in the model and use it to replace controls. This inferred in silico control is informed by the whole training data instead of a limited and biased set of control samples. Therefore, we expect it to be less noisy and present a more precise judgment of differential expression. To test this hypothesis, we apply the model to cancer samples from The Cancer Genome Atlas (TCGA) program [21]. From the negative binomial over gene counts, we can derive a *p* value for each gene in a sample and thus identify a set of significant DEGs. We first focus on analyzing breast cancer (BC) and calculating the enrichment of known cancer driver genes and subtype marker genes among the set of significant genes. This analysis is compared to a standard case–control analysis using DESeq2, which generally yields many more significant genes and much lower enrichment scores. Finally, we show that cancer driver genes are enriched among the significant genes derived from the DGD across cancers in TCGA.

In summary, our model of normal gene expression drastically improves differential expression analysis by yielding fewer false positives and extending the analysis to single cancer samples (N-of-one) *without controls*. We believe this method can significantly impact the utility of gene expression analysis, target identification, and personalized treatment.

## Results

**Fig. 1:**
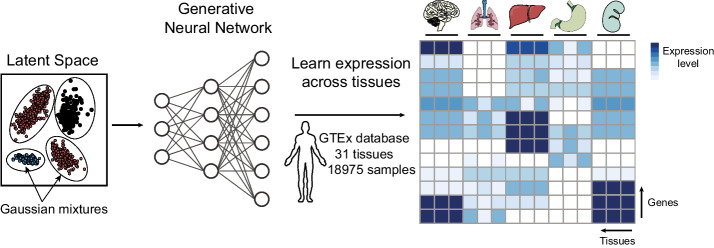
The bulk deep generative decoder. The latent space is parameterized with a Gaussian Mixture Model (left). A generative neural network trained on the GTEx database maps the latent space to the data space. Altogether, the model learns the sample representations, the mixture model, and the gene expression distribution across bulk tissues, as illustrated in the heatmap (right)

Our work aims to construct a deep generative model that learns how genes are expressed across human tissues. To limit the dimensionality of the model, we decided to use RNA-seq gene-level counts as defined in Recount3 [22] and further reduce the genes to a set of ~ 17,000 (see the “Methods” section). Our model, the DGD [20], learns a low-dimensional *representation* for every sample. After hyperparameter optimization (see the “Methods” section), we chose a representation space (or “latent space”) of dimension 50 with representations distributed according to a mixture of Gaussians with 45 components. A fully connected feed-forward decoder neural network with two hidden layers maps the latent space to sample space, resulting in a negative binomial distribution for each gene (Fig. 1). We infer the parameters of the representations, Gaussian mixture model, and decoder by training (Additional File 1: Fig. S1) our model on a stratified random subset containing 90% of the GTEx data (17,072 samples) while leaving the remaining 10% (1903 samples) as a test set (Additional File 2: Table S1).

### A generative model of gene expression for bulk samples

**Fig. 2:**
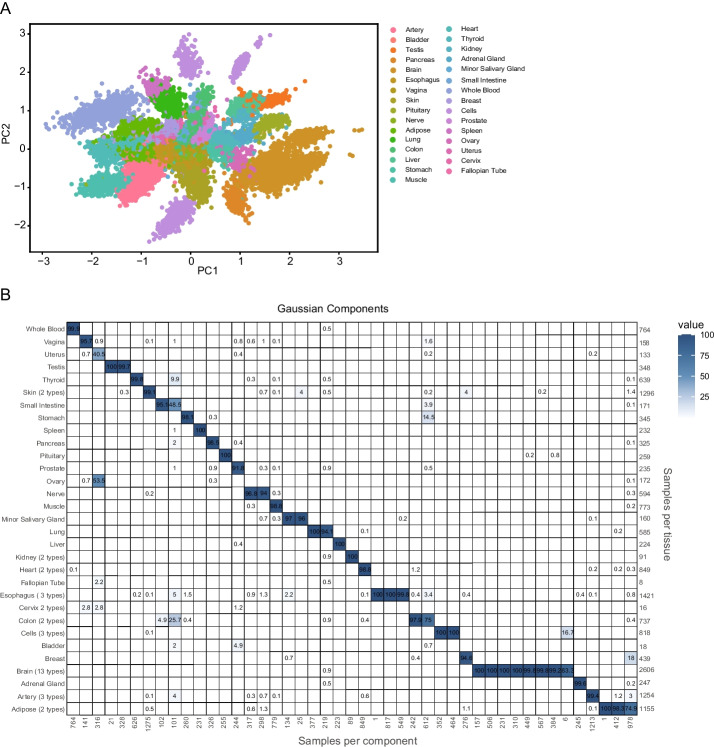
Representations for normal tissue. **A** PCA plot showing the clustering of 31 tissues in the latent space reduced from 50 dimensions to 2. Each dot corresponds to a training sample. **B** Matrix of associations showing the percentage of the samples assigned to a Gaussian component (*x*-axis) represented by each tissue (*y*-axis). The tissue types and component numbers are sorted for an optimal diagonal view. The numbers below the *x*-axis give the number of samples assigned to each component. The number of samples in each tissue is shown on the right side of the matrix

After training, we first evaluated whether the latent space of the DGD model distinguishes different tissues. A two-dimensional principal component analysis (PCA) of the latent space (Fig. 2A) shows that the DGD could find well-separated representations. The learned Gaussian mixture model (GMM) should yield a clustering of tissues based on the mixture components. To test this, we assigned each sample to the GMM component with the highest probability and evaluated how samples are distributed across components. The matrix of associations (Fig. 2B) shows a clear clustering of tissues in the GMM. The clustering of test data (Additional File 1: Fig. S2) is even clearer, but there are very few samples for some components. A single tissue dominates almost all mixture components. Exceptions to this are two components. These two are split between the uterus and ovary and small intestine and colon, respectively. Most tissues (20 out of 31) are mainly represented by only one component. Tissues modeled by multiple GMM components are typically split between two or three components. Roughly half of those tissues are represented by multiple subtypes in the data. Interestingly, the brain samples present a clear outlier in the matrix of associations, with this tissue distributed over eight GMM components.

### Finding closest-normal comparison sets for cancer samples

**Fig. 3:**
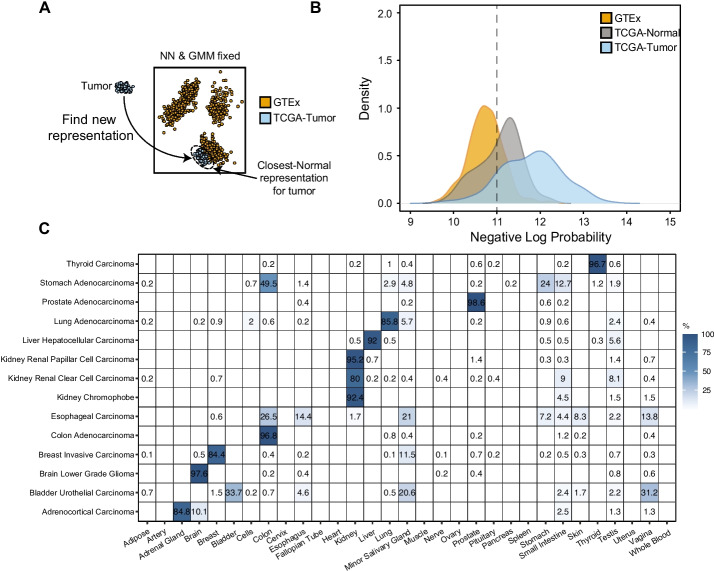
Closest normal representation for cancer samples. **A** Schematic representation of the procedure to find closest-normal representations for TCGA tumors. We find the representation by maximizing the probability of the representation while leaving the GMM and neural network fixed. **B** Density plot showing the negative log probability distributions for GTEx test, TCGA-normal, and TCGA tumor samples. **C** Matrix of associations showing the percentage of TCGA tumor samples assigned to GMM components representative of tissues. GMM components are named by their majority normal tissue type (see Fig. 2B)

Next, we evaluated whether our model of healthy gene expression could find meaningful representations for cancer samples. To do so, we used our model (trained on GTEx) to find representations for tumor samples (Additional File 3: Table S2) from The Cancer Genome Atlas dataset (TCGA). These representations were found by maximizing the probability of a representation for a cancer sample while leaving the decoder neural network and GMM parameters fixed (Fig. 3A). We interpreted this representation as the closest-normal sample to the tumor. To begin with, we evaluated the ability of our model to detect out-of-distribution examples (i.e., anomalous expression profiles) by calculating the probability of each sample given our model (Fig. 3B). As observed in Fig. 3B, TCGA-cancer samples generally gave a much lower probability than GTEx samples, while TCGA-normal samples achieved probabilities between cancer and GTEx test samples. Afterwards, we assessed whether our model matches tumor samples to the same GMM components as their healthy counterparts (Fig. 3B). We found that our model correctly assigns most tumors to their healthy normal. For 11 out of 14 tissues, more than 80% of the samples are assigned to their corresponding normal tissues. The three tissues with the lowest classification accuracies are the bladder, stomach, and esophagus.

### Detecting cancer differentially expressed genes without controls

**Fig. 4:**
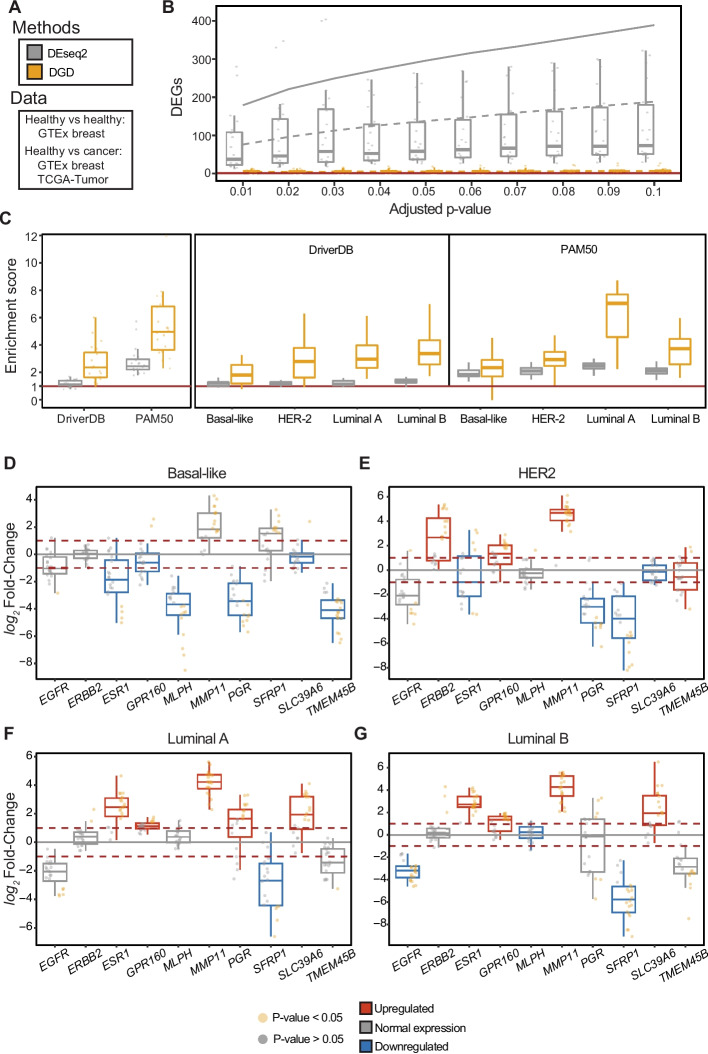
Differential expression analysis of Breast cancer and its subtypes. **A** Schematic overview of the samples used in our experimental set-up. **B** “Negative control” experiment comparing healthy test samples against the DGD (yellow) and controls for DESeq2 (gray). Twenty random test samples were chosen (shown as dots) and summarized by the boxplots and the mean (dashed gray line) for different cut-offs on adjusted *p* value (*x*-axis). As controls for DEseq2, we used the whole training set of breast tissue. We also tested with a random subset of 5 controls and show the mean as the solid gray line. **C** Enrichment score across breast cancer subtypes for driver genes and PAM50 genes derived from DGD and DESeq2. 20 random cancer samples were selected and compared to the DGD (yellow) and to DEseq2 using GTEx breast samples as controls (gray, see the “Methods” section for sample selection). The enrichment of cancer driver and PAM50 among the significant genes (adjusted *p* value < 0.01) is shown for each breast cancer subtype. **D**–**G** Breast cancer-specific differential expression analysis on a subset of ten marker genes, using 20 randomly selected samples. The box plots are colored based on whether the gene is known to be differentially expressed (red: upregulated; gray: normal expression; blue: downregulated) in a cancer subtype. The dots are colored based on the adjusted p value obtained by the DGD in each replicate

We extended the DGD to detect differentially expressed genes. We performed a two-tailed negative binomial test for the distribution generated from the closest-normal representation (see the “Methods” section). Our analysis focused on breast cancer (BC), as both TCGA and GTEx contain a large number of samples (Fig. 4A). We first evaluated the specificity of our approach in a normal vs. normal “negative control” experiment. Afterwards, we tested one cancer sample against the model (N-of-one) to resemble common clinical settings. We compared our approach in both “negative control” and “positive control” to a standard analysis of one sample against GTEx normals using DEseq2 [11], a widely used statistical method to detect differentially expressed genes.

We assessed the model’s specificity in a normal vs. normal analysis using healthy breast tissue from GTEx. It is assumed that there should be no or very few DEGs when comparing normal samples. Therefore, the number of DEGs here functions as the number of false positives and is thus related to the specificity of the model. We randomly selected 20 samples from the breast test set (out of 42 GTEx samples) and compared each one against the 440 control samples from the training set using DEseq2. We used the same 20 random samples and compared each one to the DGD model. The number of significant genes found by the two methods are shown in Fig. 4B for varying adjusted *p* values. We performed the same analysis using five randomly selected samples from the training set as the comparison set (full line in Fig. 4B). Ideally, this analysis should return no significant genes. However, DEseq2 found many DEGs, calling on average 76.15 genes (adjusted *p* value < 0.01 and absolute log2 fold change > 1) using the whole GTEx and 179.05 when using 5 random samples with 20 repetitions as controls. The DGD, on the contrary, found almost no false positives with an average of 4.25 called genes (using the same filtering criteria as DEseq2).

To compare the sensitivities of DGD and DEseq2 in a “positive control” experiment, we analyzed their ability to correctly identify marker genes known to be important in BC. Two sets of BC-related genes were curated for this purpose: (I) driver genes from the DriverDBv3 database [23] and (II) the PAM50 [24] set of subtype-specific BC genes (the “Methods” section). As a metric, we calculated a gene enrichment score, the fraction of the number of BC-related genes among the significant ones divided by that expected by random chance (total number of BC genes divided by number of all genes). We use this measure to ensure that a method cannot score high by simply predicting a very large number of differentially regulated genes. The higher the enrichment, the more the method agrees with a set of marker genes. A value of 1 means no enrichment.

We evaluated enrichment scores across four PAM50 breast cancer subtypes: basal-like, HER2, luminal A, and luminal B that we annotated in TCGA from a previous study [25]. For this analysis, we applied clinical filters to ensure greater homogeneity of samples (Additional File 4:Table S3). We performed experiments similar to those described above, randomly selecting one sample (20 repetitions) and comparing it to the 143 GTEx breast samples (40–70-year-old females) and the model for DEseq2 and DGD, respectively. The DGD obtained higher enrichment scores than DEseq2 for all subtypes regarding driver genes (DriverDBv3) and PAM50 genes (Fig. 4C). The DGD obtained an average enrichment score of 3.46 and attained a particularly high score for the luminal A subtype in the PAM50 marker set. The DEseq2 average score was 1.71, and it was lower than the model for subtypes. To ensure that the results were not due to a too restrictive filtering, we performed the same analysis without any filtering in the whole TCGA breast cancer dataset (Additional File 1: Fig. S3). We found that our filtering scheme did not affect the enrichment scores (Additional File 1: Fig. S3A). We also explored whether some of our filters (age, tumor purity, necrosis etc.) had any impact on the enrichment scores, finding, unsurprisingly, that a higher tumor purity is associated with a higher enrichment score in marker genes (Additional File 1: Fig. S3B).

Next, we selected a subset of PAM50 genes to evaluate whether the DGD captured the expression differences between subtypes. We focused on genes that achieved a significant *p* value in at least 10 replicates of the PAM50 experiment performed above (Additional File 5: Table S4), leading to ten genes across the four subtypes. For each gene, we evaluated whether the DGD detected differential expression (i.e., a significant p value) and the expression trend (i.e., upregulation or downregulation) as defined in the PAM50 set [26–29]. The DGD correctly identified 32 of the 40 expected gene expression patterns (Fig. 4D–G), obtaining similar performances across the subtypes. The best overall performance was achieved for the luminal A and luminal B subtypes (eight out of ten genes determined correctly). The lowest performance was for basal-like and HER2 subtypes (six out of ten genes correctly determined). Summarizing across genes, *ERBB2*, *GPR160*, and *PGR* have the expected trend in all subtypes. *ESR1*,* SFRP1*,* MLPH*, and *MMP11* have the expected trend in three out of four subtypes. The DGD only under-performed on *EGFR*,* SLC39A6*, and *TMEM45B*, which were wrongly identified in two out of four, two out of four, and three out of four subtypes, respectively.

**Fig. 5:**
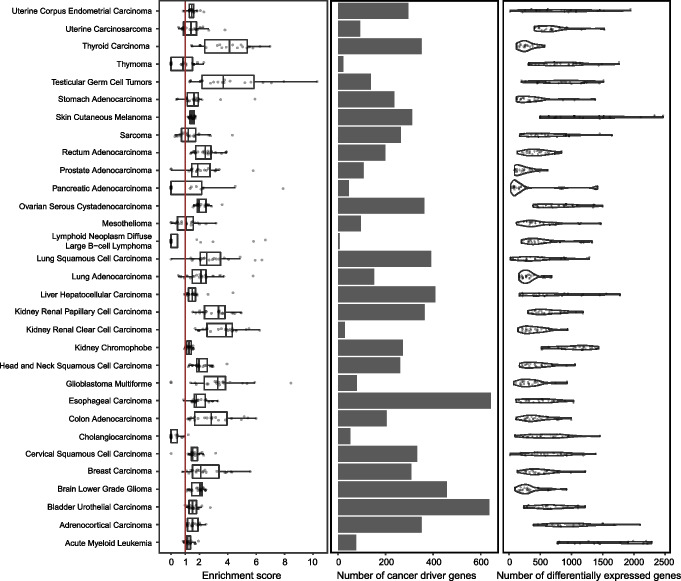
Differential expression and enrichment analysis for cancer driver genes across TCGA. A single sample was randomly selected from each TCGA-tumor type to calculate the differentially expressed genes with our model. This process was repeated 20 times. The left panel shows boxplots of the enrichment scores for every TCGA-tumor. Dots indicate the experiment replicates. The middle plot shows the number of cancer driver genes present in CancerDBv3 for each cancer. The right panel shows a violin plot of the number of differentially expressed genes in every replicate of our experiments

As a final analysis, we extended our model to all the 31 TCGA cancer types to better estimate the DGD’s general performance (Fig. 5). As described above, we performed N-of-one experiments using 20 repetitions, and we evaluated the enrichment score of the DriverDBv3 gene set for each cancer type (Additional File 6: Table S5). The DGD found more cancer marker genes than expected by chance for all cancer types (mean enrichment score 2.05, mean range 0.24–4.70). Specifically, the scores were higher for kidney renal clear and papillary cell carcinomas (mean enrichment of 3.65 and 3.18, respectively), thyroid carcinoma (mean enrichment 4.00), and testicular germ cell tumors (mean enrichment 4.70). Cancers with median enrichment below 1 tend to have a small number of cancer driver genes in the database (thymoma, pancreatic adenocarcinoma, lymphoid neoplasm diffuse large B cell lymphoma and cholangiocarcinoma).

## Discussion

A lot of attention is currently directed towards single-cell RNA sequencing due to its potential higher resolution and recent advances in its scalability. However, bulk sequencing is still the main work-horse for clinical use [30]. Differential expression analysis between disease and normal has relied on solid statistical methods but is challenged by the difficulty in obtaining suitable control samples and large enough sample sizes. Here, we introduce a method requiring no biological replicates or matched controls. The model we present learns the gene expression of normal tissue samples and generates an in silico normal sample closest to the disease sample at hand.

To assess whether the model learns meaningful representations, we analyzed how well GMM components model tissues as the most prevalent biological feature in the data. The model clusters representations well in a tissue-dependent manner and typically assigns only one to a few Gaussian mixture components to each tissue type. Most tissues not being modeled by a single component are represented by multiple subtypes in the data or present complex biological tissues, such as the brain. Since the number of components in the GMM is much higher than the number of tissues, it is understandable that multiple components model more complex structures. This may also explain why under-represented tissues from larger systems are clustered together. An example is the component gathering samples from the uterus, ovary, fallopian tube, and cervix. Given that the representations are found in a completely unsupervised fashion, we find the clustering to be remarkably interpretable.

In addition to providing well-clustered representations, the model of normal gene expression can replace control samples in the differential gene analysis of disease. This is achieved by finding the closest normal representation in the model. This representation is used to compute a probability distribution for the expression counts of each gene. We calculated the total probabilities of the GTEx test, TCGA normal, and TCGA cancer samples. We found that the probabilities derived from the model of normal are highest for GTEx test data and decrease strongly for TCGA cancer as expected. Probabilities for TCGA normal samples lie between the two, which may be explained by previous findings suggesting that adjacent normal tissue carries cancer traits [6]. A similar analysis has previously been performed using a Bayesian approach [10], but the separation seems much clearer [10], although a direct comparison is difficult. When analyzing the quality of the integration of cancer samples into latent space, we observe that most representations of cancer samples are consistent with the tissue of origin. For most cancers, more than 80% of samples end up in the expected tissue component. These results are comparable to [10]. Here it is worth noting that we do not necessarily expect a perfect match to the tissue of origin because, in metastatic cancers, the tumor may be more similar to the tissue of origin.

It is difficult to assess the performance of differential gene expression analysis without knowing the ground truth. We have therefore used an enrichment score for known cancer genes among the significant genes to compare our model to a standard analysis using breast cancer as a case. Firstly, we assessed the specificity of our model and DESeq2 by comparing normal vs. normal as a negative control. While DESeq2 yields large numbers of significant genes (1.75% of all genes, given a log2-fold change > 1 and an adjusted *p* value < 0.05), here interpreted as false positives, our model reports only 0.03% of the genes to be differentially expressed under the same conditions. Secondly, we calculate the enrichment of relevant known cancer genes among the significant genes derived from differential expression analysis of breast cancer samples. In this positive control, we used a set of known breast cancer driver genes and a PAM50 set of genes discriminating between breast cancer subtypes. In the general breast cancer case, we see consistently higher enrichment when using the DGD compared to DESeq2. This is also true, for the most part, for the cancer subtypes. However, there are some discrepancies with respect to the expected driver genes. For instance, according to our model, *TMEM45B* should be upregulated in the *HER2* subtype but is downregulated on average. Yet, we do not expect a perfect concordance between the PAM50 panel and tumor samples, as many patient-specific factors can affect gene expression. Altogether, our evaluation of breast cancer shows that the DGD returns much fewer false positives and a higher proportion of truly relevant genes compared to DESeq2. More importantly, we expect our model to improve the classification of different cancer subtypes. Subtype classification is performed based on which genes are differentially expressed, and the ability to better detect differential expression should improve classification.

The method presented is using RNA-seq counts for a selected subset of about ~ 17,000 genes from GTEx bulk samples and is of course limited to analyses involving these genes in similar samples. The methodology, however, can be used more generally and it may for instance be possible to extend it to analysis of transcripts, small RNAs, and other data types.

## Conclusion

The model introduced and discussed here presents an important step towards using bulk gene expression analysis for precision medicine. Given that the DGD does not require control samples, the potential impact for differential expression analysis with *single disease samples* is immense. We have tested the method on cancer, but there is no reason that it should not perform as well on other diseases. Because of the impressive performance on even single samples, we believe our model has a high potential for application to rare diseases. Since the differential expression analysis generally results in much fewer false positives than in a standard analysis, it will enable us to find genes that are truly involved in disease. This can, in turn, increase the possibility to find druggable targets and to understand diseases on an individual level much better than it has previously been possible with bulk data.

## Methods

### The model

#### Architecture and hyperparameters

The full model consists of a Gaussian mixture model as the parametrized distribution over latent space and a decoder as presented in [20]. We performed hyperparameter optimization on the latent dimension, number of hidden layers in the decoder, and number of Gaussian components. Representations were explored with dimensions between 10 and 100. We further tested various decoder architectures with two to four hidden layers of 200 to 10,000 hidden units. For the Gaussian mixture model, we tried different distribution complexities with 20 to 50 components. Each representation of a sample received a 50-dimensional vector initialized with zero. The decoder consists of a 50-dimensional input layer fed with the representations, two hidden layers, and an output layer with units corresponding to each of the genes in the data. The two hidden layers are of size 500 and 8000, respectively, immediately followed by a ReLU activation [31, 32]. The output values are transformed into negative binomial distributions over expression counts for each gene. The negative binomial is given by:

$$NB\;(k;\;m,\;r)\;=\;\frac{\Gamma(k+r)}{k!\Gamma(r)}{\;(\frac r{r+m})}^r\;{(\frac m{r+m})}^k$$

where *k* is the library size, *m* is the mean, and *r* the dispersion parameter. The decoder outputs are passed through ReLU activation and scaled with the sample gene expression mean and used as the mean (*m*) of the distributions. The trainable dispersion parameters (*r*) of the negative binomials are gene-specific and initialized with 2. The GMM consists of 45 mixture components. The priors are a mollified uniform distribution (the “softball” prior) with a spread of 7 and a sharpness of 10 for the means, a Gaussian with a mean of 1 (corresponds to a standard deviation of 0.1), and a standard deviation of 1 for the negative logarithmic diagonal covariance and a Dirichlet distribution with all alphas equal to five. For more details, please refer to [20].

#### Training

The model was trained for 200 epochs with a batch size of 256. The optimizer of choice was Adam [33] without weight decay and betas 0.5 and 0.9. Because the representations are updated every epoch, the decoder, representation, and GMM received distinct optimizer instances with learning rates 10^−4^, 0.01, and 0.01, respectively.

#### Evaluation

Representations for single test samples are optimized as described in [20] with all model parameters fixed. For a new datapoint, representations were initialized from the component means. This results in 45 representations per sample. These were optimized with the fixed model for 10 epochs, after which the best representation is selected for each sample. To perform differential expression analysis, we further optimized the selected representation for another 50 epochs.

#### Differential expression

We used the DGD to find differentially expressed genes for tumor samples. An optimal representation is found for each tumor sample as described above. These representations are interpreted as the closest-normal for each tumor—an in silico normal. In our setup, we want to test if counts for a given gene are significantly different between the tumor sample and the normal tissue output of the neural network. Let $NB\;({k;m}_i,r_i)$ be the negative binomial distribution for gene $i$ and ${x}_{i}$ be the actual count in the tumor sample. We test the hypothesis that the observation from the tumor is from the in silico normal. Therefore, the *p* value for ${x}_{i}$ is the sum of all counts with a probability lower than that of ${x}_{i}$:

$$p\;\mathrm{value}\;=\;\sum\nolimits_{k=0}^K\;NB\;(k;\;m_i,\;r_i)\;I\;\lbrack NB\;({k;\;m}_i,\;r_i)\;\leq\;NB\;(x_i{;\;m}_i,\;r_i)\rbrack,$$

where *K* is the library size and *I* is an indicator function taking a value of 1 when the condition is fulfilled and otherwise 0. The above expression yields an exact *p* value for the negative binomial distributions. However, it requires summing over all read counts across genes. For the sake of efficiency, we therefore obtain an asymptotic p value by summing over an evenly spaced grid of 10^4^ in the domain of $K$.

### Data

#### Data collection and processing

The raw gene count expression data from the Genotype-Tissue Expression (GTEx) and The Cancer Genome Atlas (TCGA) were downloaded from the Recount3 platform [22] (https://rna.recount.bio/) using the built-in R packages. Additionally, the sample metadata files were also acquired from Recount3 [22].

At the time of download (February 9, 2022) there were 31 different tissue types in GTEx and one group without tissue information, with a total of 19,214 individual samples (Additional File 2: Table S1). The 133 samples without tissue information were removed, and duplicates were dropped before further processing. We performed feature selection using the *filterbyExpr* [34] function with default parameters in R (edgeR version 3.34.1) to remove lowly expressed genes. We further reduced the set of genes to protein-coding genes according to the UCSC Genome Browser [35]. The gene list is available in our GitHub repository (see below). After preprocessing, the GTEx dataset contained 18,975 samples and 16,883 annotated protein-coding genes. We have deposited the gene list used to train our model in Zenodo with https://doi.org/10.5281/zenodo.10021626 [36], and it is released under the GNU license. The gene list can also be found in Zenodo with the link: https://zenodo.org/records/10021626 [36].

For training and evaluation, the samples were split into a train and a test set, with the test set representing roughly 10% of the data. Sampling was performed in a proportional stratified random manner, meaning that 10% of the samples were randomly chosen and assigned to the test set for each tissue. This procedure resulted in 17,072 train and 1903 test samples.

As for cancer data, The Cancer Genome Atlas (TCGA) Program provided 33 different cancer types. We selected the set of genes present in our processed GTEx dataset and separated the TCGA samples into a normal adjacent set (747 samples) and a tumor set (10,601 samples) following the metadata file.

#### TCGA tissue selection

For an assessment of the model’s capability to assign independent samples to the correct tissue clusters according to the training data, we selected 14 cancers from TCGA that fulfilled the following two conditions: (1) the tissue corresponding to the cancer is present in GTEx and (2) the tissue must have at least ten adjacent normal samples from each cancer type [37]. We also included the adrenal and brain tumor samples, even though they lack adjacent normal samples, to compare our result with the work of Vivian et al. [10]. This results in 6111 TCGA tumor samples and 624 TCGA adjacent normal samples (Additional File 3: Table S2).

In applying our differential expression analysis to cancer types other than breast cancer, we include TCGA cancer samples from the 31 cancer types overlapping with those found in DriverDBv3 [23].

#### TCGA breast cancer subset

To obtain a homogenous breast cancer (BC) dataset for testing, we curated the TCGA BC samples to only include primary tumors from women between 40 and 70 years of age. We excluded samples with low tumor cell percentage (defined as < 50%) or a high level of necrosis (defined as > 5%), in addition to samples from patients with known metastasis, stage IV or stage X tumors, or prior cancer diagnosis. For a full list of selection criteria and columns from metadata used for curation, see Additional File 5: Table S4. The number of available BC samples for analysis was reduced from 1256 to 381.

#### Cancer driver genes and PAM50 genes

We downloaded a list of cancer driver genes for each cancer type from DriverDBv3 [23], and we matched the symbol names in the database to the Ensembl IDs from recount3. The PAM50 gene set used for BC subtype classification (basal, luminal A, luminal B, and Her2-enriched) [24] was downloaded through the R-package *genefu* [27]. As our model testing and comparison with DEseq2 pertained to the expression profile of BC subtype tissue vs. normal tissue (i.e., not between subtypes), we filtered the PAM50 dataset to only include the genes which were specific to a single subtype or which could distinguish BC subtype(s) from normal tissue. We noted the expected directionality of each of the PAM50 genes (upregulated or downregulated) for a contrast. The selection of genes was based partly on literature [26, 28, 29] and partly on the robust normalized PAM50 scores [27]. The PAM50 gene set was reduced to 34 genes.

### Analysis

#### Evaluation of tissue specificity

Clustering performance according to tissue type was evaluated based on the GMM probability densities for each sample’s representation. For this purpose, samples are assigned the GMM component that achieves the highest probability density for their inferred representations. We calculated the percentage of each tissue per component as the number of samples of a given tissue clustered in a given component divided by the total number of samples assigned to this component.

$$percentage\;=\;\frac{\#\;of\;tissue\;-\;specific\;samples\;clustered\;in\;component}{\#\;of\;total\;samples\;in\;component}\;x\;100$$

This is done independently for the train set (Fig. 2B) and test set (Additional File 1: Fig S2).

#### Matching TCGA to normal tissues

We use the TCGA data described above to evaluate the mapping of unseen and independent data onto the latent space. New representations for all 6111 TCGA tumor samples were found using the DGD trained on GTEx data. The resulting GMM probability densities for the TCGA representations are used to evaluate how well new samples match the correct tissues of the training representation. We define the “correct” tissue as the tissue that best represents a given GMM component. Our evaluation metric is the percentage of TCGA samples of a given tissue matched to the corresponding GMM component with respect to the total number of TCGA samples for this tissue.

$$\%\;TCGA\;in\;correct\;clusters\;=\;\frac{\#\;of\;TCGA\;-\;tumor\;samples\;in\;"correct"\;component}{\#\;of\;TCGA\;-\;tumor\;samples\;of\;tissue\;in\;total}\;x\;100$$

Bladder samples were evaluated differently due to the lack of a bladder-specific component in the normal model. Instead, we evaluated a correct match as TCGA and GTEx bladder samples were assigned to the same component(s).

#### Comparing GTEx and TCGA gene expression predictions

The total probability of a sample is the product of the probabilities across all 16,883 genes given by the model (also used in the model likelihood). We calculate the negative log-probability mass of each sample for three datasets: GTEx test, TCGA adjacent normal, and TCGA tumor. For this comparison, we use subsets containing ten tissues: adrenal, brain, breast, colon, kidney, liver, lung, prostate, stomach, and thyroid. We apply this analysis for a pan-tissue comparison based on the eight tissues only, because adrenal and brain are missing in the TCGA adjacent normal subset. For a fair comparison, we ensure equal samples for a given tissue across the three datasets. If all datasets have more than 20 samples for a given tissue, we randomly select 20 samples from each subset for that tissue.

#### Differential expression analysis in TCGA breast cancer

Differential expression analysis (DEA) performed by our model is compared to DESeq2 by the enrichment of cancer driver genes among DEGs.

For a general comparison, we perform 20 single-sample experiments using 1 random breast cancer sample (case) from the population of 40–50-year-old Caucasian females. This procedure leaves us with 166 samples. Genes that result in absolute log2-fold changes greater than one and adjusted *p* values below 0.01 are accepted as differentially expressed. The enrichment score is then given as the normalized number of DEGs belonging to the group of breast cancer driver genes or PAM50 genes, respectively.

$$ES\;=\;\frac{\mathrm{enriched}\;\mathrm{cancer}\;\mathrm{marker}\;\mathrm{genes}\;\ast\;16,883\;\mathrm g\mathrm e\mathrm n\mathrm e\mathrm s}{\mathrm{significant}\;\mathrm{genes}\;\ast\;\mathrm{cancer}\;\mathrm{marker}\;\mathrm{genes}}$$

We perform a comparable DEA with DESeq2 using all 44 GTEx samples (control) under the same conditions (40–50-year-old females).

We also perform single-sample analyses of the four available breast cancer subtypes: basal-like (84 samples), HER2 (37 samples), luminal A (176 samples), and luminal B (84 samples), both for our model and DEseq2. We randomly choose one sample from each of the four subtypes as a case sample and used all GTEx breast tissue samples (40–70-year-old females, 143 samples in total) as controls in the DEseq2 method. The experiment is repeated 20 times for each subtype.

#### False positive analysis

In order to assess the false positive rates of the methods, we selected a random GTEx breast sample from the test set (42 samples) as a false case sample. We performed DEA with our model and DESeq2 to yield false positive DEGs for adjusted *p* values ranging from 0.01 to 0.1 (absolute log2-fold change greater than 1). We performed this 20 times and reported the resulting DEGs as false positives. As controls for DESeq2, we used all breast samples from the GTEx training set (440 samples) as controls. We also perform the analysis for DEseq2 by choosing five controls, which are randomly selected from the breast samples mentioned above.

For the enrichment analysis of Cancer Driver Genes for multiple cancer types, 31 different cancer types are used: adrenocortical carcinoma, bladder urothelial carcinoma, brain lower grade glioma, breast carcinoma, colon adenocarcinoma, kidney cancer (kidney chromophobe, kidney renal clear cell carcinoma, kidney renal papillary cell carcinoma), liver hepatocellular carcinoma, lung adenocarcinoma, prostate adenocarcinoma, stomach adenocarcinoma, and thyroid carcinoma. We perform 20 single-sample experiments for each of the cancer types. For each cancer type, its respective cancer driver gene list was used in the enrichment score calculation.

### Supplementary Information

**Additional file 1: Fig S1.** Loss curves. **Fig S2.** Matrix of associations for the GTEx test set. **Fig S3.** Enrichment scores for TCGA Breast data.

**Additional file 2: Table S1.** Overview of the samples from GTEx.

**Additional file 3: Table S2.** Overview of TCGA tumor and TCGA adjacent samples.

**Additional file 4: Table S3.** Clinical filters for breast cancer subtypes.

**Additional file 5: Table S4.** Expression trend of the selected 34 PAM50 genes and the number of times it was found to have a significant differential expression.

**Additional file 6: Table S5.** Number of cancer driver genes.

**Additional file 7.** Review history.

## Data Availability

The source code is released under the GNU license and it is freely available at https://github.com/Center-for-Health-Data-Science/bulkDGD [36]. The repository contains the source code, command-line utilities, and tutorials to facilitate its usage. Furthermore, we provide easy install guidelines through the Python Package Index and conda. We have deposited the version of the software used in the manuscript in Zenodo with https://doi.org/10.5281/zenodo.10021626 [36]. The data used in this study is available in the recount3 database [22] and The Cancer Genome Atlas database [21]. The results of the differential gene expression analysis generated in this study are available at Zenodo with https://doi.org/10.5281/zenodo.10026219 [38]. The gene list used to train the model is available at Zenodo with https://doi.org/10.5281/zenodo.10021626 [36]. The code used to perform the analysis and train the model is available at Zenodo with https://doi.org/10.5281/zenodo.10021626 [36].
