# Supplementary material for: N-of-one differential gene expression without control samples using a deep generative model
Source: Genome Biol. 2023 Nov 16;24:263. doi: 10.1186/s13059-023-03104-7 (PMC10655485; doi:10.1186/s13059-023-03104-7)
Supplement: Supplementary file 1 — Additional file 1: Fig S1. Loss curves. Fig S2. Matrix of associations for the GTEx test set. Fig S3. Enrichment scores for TCGA Breast data. [file 13059_2023_3104_MOESM1_ESM.pdf]

Supplementary Figures for

A generative model of normal tissue gene expression enables differential expression in cancer with one sample

Iñigo Prada-Luengo[1,†], Viktoria Schuster[2,†], Yuhu Liang[1,†], Thilde Terkelsen [2], Valentina Sora[1] and Anders Krogh[1,2,*]

[1]Department of Computer Science, University of Copenhagen, Denmark
[2]Center for Health Data Science, University of Copenhagen, Denmark
[†]equal contributions

Corresponding author: Anders Krogh, email: akrogh@di.ku.dk

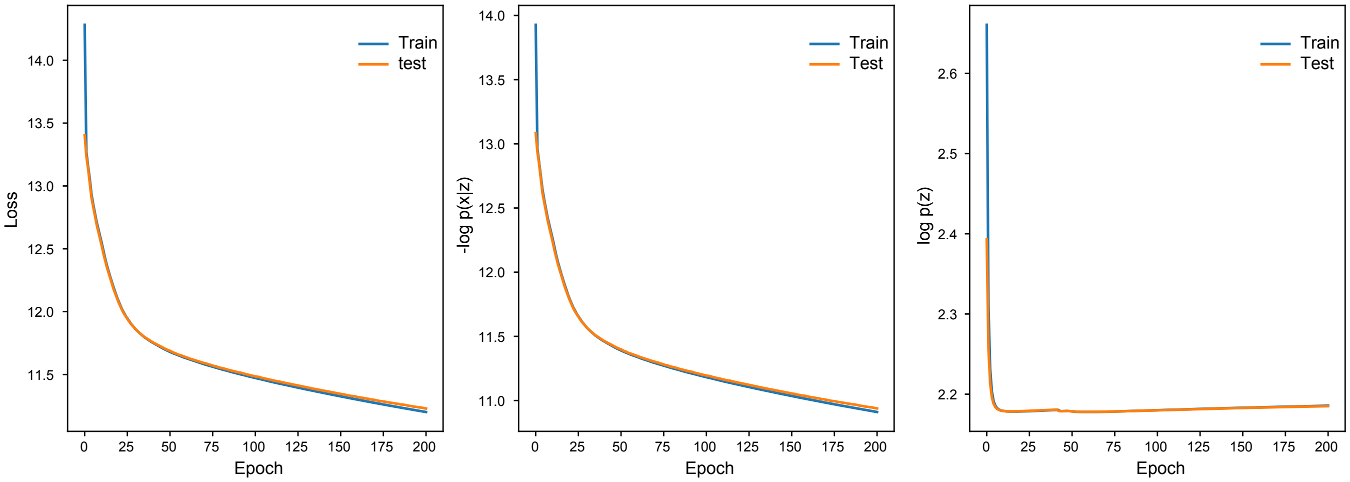

Fig S1: Loss curves. The figure shows loss curves for training and test sets in blue and orange, respectively. The left panel shows the total loss curve for the DGD model. The two panels on the right separate the loss into two terms. The middle panel shows the reconstruction loss of the DGD, and the right panel shows the Gaussian mixture model loss.

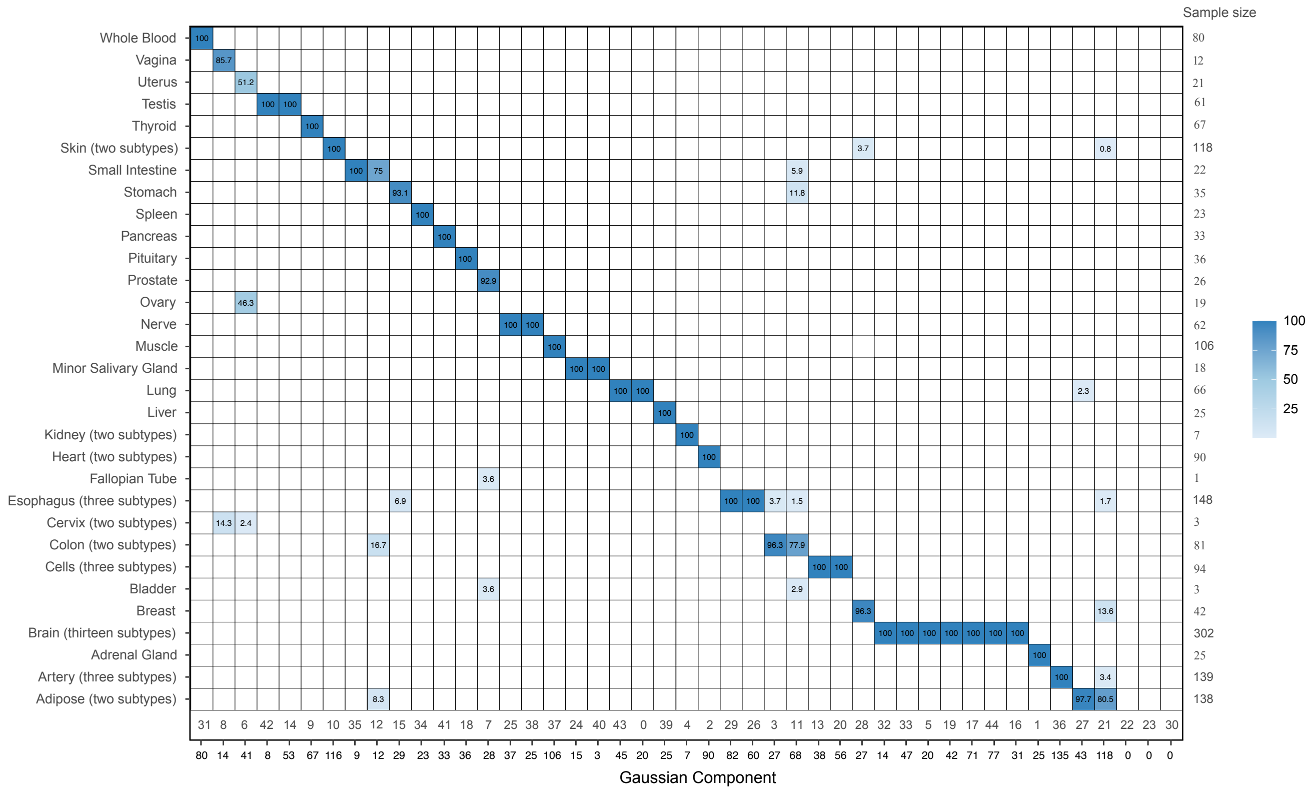

Fig S2: Matrix of associations for the GTEx test set. The matrix shows the percentage of samples assigned to the Gaussian components (x-axis) represented by each tissue (y-axis). The tissue types and components have the same order as in Figure 2B. The row inmediately below the matrix shows the indexes of the Gaussian components. The row below shows the number of samples in each component. The column on the right shows the number of samples in each tissue.

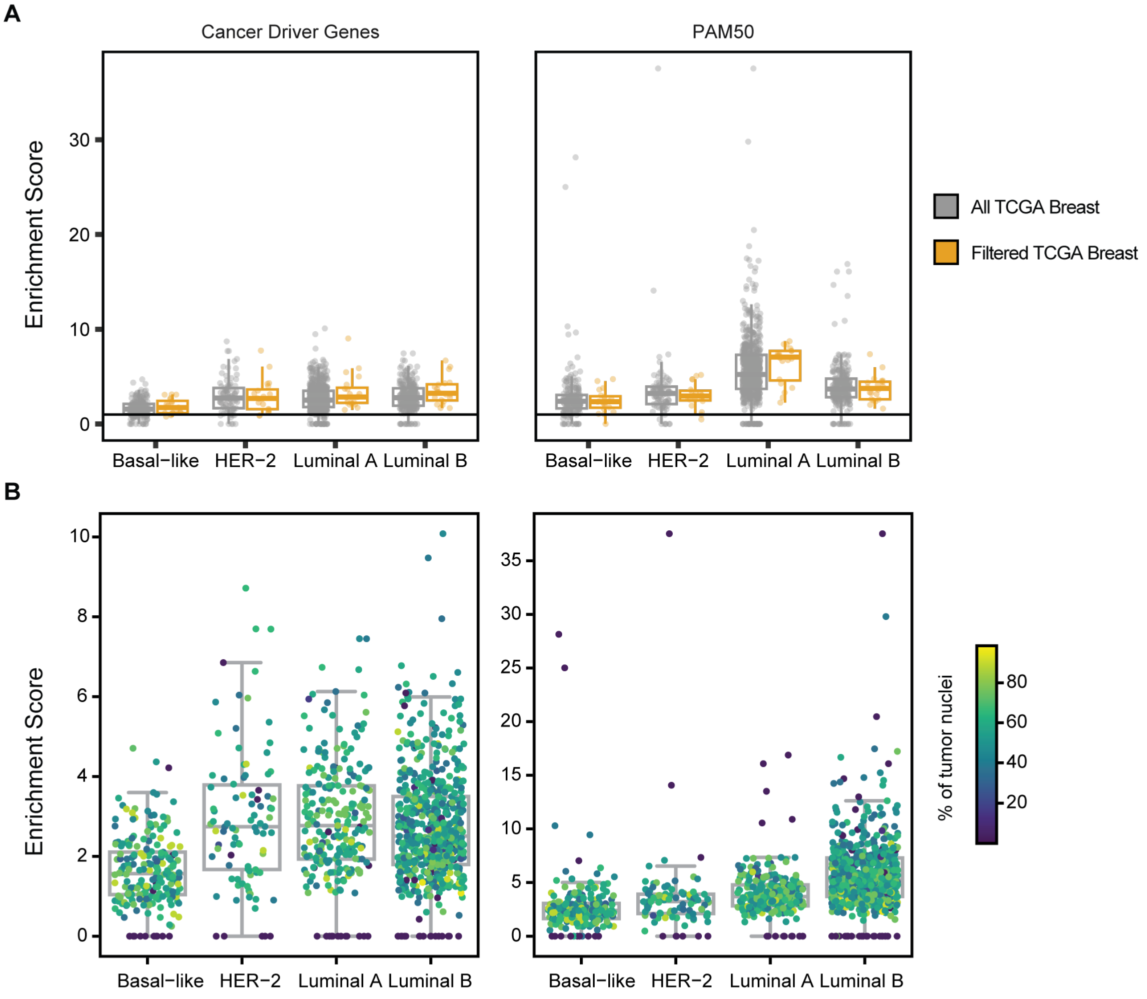

Fig S3: Enrichment scores for TCGA Breast data. **A** Enrichment scores for All TCGA breast cancer samples (n=1168) and the filtered data (as in Figure 4C) to ensure homogeneity of the samples (Supplementary table S3). **B** Enrichment scores for all TCGA breast cancer samples with data points colored as a function of tumor purity.
