# Supplementary material for: N-of-one differential gene expression without control samples using a deep generative model
Source: Genome Biol. 2023 Nov 16;24:263. doi: 10.1186/s13059-023-03104-7 (PMC10655485; doi:10.1186/s13059-023-03104-7)
Supplement: Supplementary file 7 — Additional file 7. Review history. [file 13059_2023_3104_MOESM7_ESM.docx]

Review History

**First round of review**

**Reviewer 1**

**Were you able to assess all statistics in the manuscript, including the appropriateness of statistical tests used?**

No.

**Were you able to directly test the methods?**

No.

**Comments to author:**

The analysis of differential gene expression using bulk RNA sequencing data is important for diagnosing, predicting outcomes, and understanding diseases. But, often suitable control samples are missing. In the current manuscript, researchers propose a deep generative decoder model (DGD) based on [Schuster&Krogh, 2021+2022] that can substitute control samples by a so called generated "in silico closest normal sample". The model is trained on RNA-seq data from healthy tissues and learns a latent representation that effectively groups tissues together.

The conclusions of the paper are adequately supported by the data shown: the paper demonstrates how the proposed approach successfully identifies cancer driver genes and marker genes specific to different subtypes of breast cancer with a high degree of specificity. The method outperforms a current state-of-the-art method DESeq2 in detecting differentially expressed genes. Genes identified using their model show significant enrichment for cancer-specific driver genes for most types of cancer in TCGA.

Interestingly, the in silico closest normal sample performs better than actual "normal" samples from the same cancer patients in TCGA data. Thus, the approach is a good alternative for DESeq2-based differential expression analysis, in particular for the quite relevant case that no normal control samples are available, i.e. for cancer samples of patients.

I think the claims are somewhat far reaching, but as claimed in the paper quite well supported by a number of assessments and in-silico experiments. These experiments are clearly described in the paper and yield supportive results.

Sufficient details of the methods and evaluation are nicely described. It appears that the results could be reproduced.

But, the actual reproduction of results is difficult. Code and models are available via a github repository mentioned in the manuscript. The documentation and application of the model could be made much easier and be much better documented in order to apply and reproduce the results/figures of the paper.

The DGD model and its training requires more scripts and data described in previous papers of the authors. I am confident, that the model is trained and performs as described in the paper, but it is difficult to reproduce it from the tools provided.

In order to be a useful alternative to standard applications of DESeq2 a better tutorial or scripts to apply the DGD model for the differential analysis of new samples is required.

The method is likely of broad utility: as mentioned above, the method is somewhat superior to the very widely used (in fact, the de-facto standard tool) DESeq2 applied with control samples. The method has better performance and can be applied when no controls are available or when control samples have not been measured.

The paper is of interest to a broad audience of biologists: The proposed method is a better alternative to DESeq2, a standard tool typically applied for differential gene expression analysis (the DESeq2 paper published in Genome Biology is cited more than 50.000 times).

But it should be made much clearer how a broad audience could make easy use of the DGD model for standard DE analyses.

Scripts should be provided to reproduce the model, the data and/or the figures. The documentation of provided code and data should be improved in order to fulfill the claim of "a high potential for application" to (other/rare) diseases the application of deriving and using the DGD models should

be much better supported by respective documentation, scripts and use cases.

**Reviewer 2**

**Were you able to assess all statistics in the manuscript, including the appropriateness of statistical tests used?**

No.

**Were you able to directly test the methods?**

No.

**Comments to author:**

The authors propose a novel approach to circumvent the problems and limitations of differential analysis of expression data. To date, the published methods lack reproducibility, making them more difficult to interpret and use in clinical practice. One of the difficulties in the gene differential analysis is the problem of the controls. The authors present a deep generative model (DGD) based on a previously developped method deposited in ArXiv (Schuster and Krogh 2022). The Deep Generative Decoder (DGD) is "a simple generative model that computes model parameters and representations directly via maximum a posteriori (MAP) estimation", when trained with bulk RNAseq data it returns a small-sized representation.

Here, the novelty is to propose an application of the DGD model in the analysis of tumor RNAseq data and the possibility of the "gene expression model of normal tissue to replace control samples and to enable differential expression" .

The authors' proposal is interesting for several aspects, strengthening the methods of analysis of RNAseq to facilitate its use in the clinic and offering the possibility of using the numerous public data to enrich and improve analyzes

However some questions need to be discussed, they mainly concern the limit of the approach.

1/ Can the author discuss the limit of the method starting from annotated genes or "raw gene count" from a reference. Rnaseq is a technology with a high potential to identify a great transcript diversity not fully accounted for even by the best reference databases. Each RNA is the result of a complex chain of events that combines by itself genetic variation, transcription regulation and RNA processing.

To perform their analysis, the authors used the "raw gene count expression data from the Genotype-Tissue Expression (GTEx) and The Cancer Genome Atlas (TCGA) (downloaded from the Recount3 database (https://rna.recount.bio/)...". ( methods p11).

Can the author precise the reference and comment its limit ?

2/ The second concern the GTEX cohort and the representation of cell subtypes. GTEx is an interesting dataset containing samples from 31 different human tissues. However each tissue, often originating from an organ in the Gtex cohort, is by itself a complex representation of a mixture of cell populations. A tumor type is a complex representation of the tissue and as such a complex mixture of cell types. Breast cancer is a good illustration of this diversity with Basal, Luminal A, Luminal B and Her2-enriched subtypes.

In methods p12, the authors indicate : "To obtain a homogenous breast cancer (BC) dataset for testing, we curated the TCGA BC samples to only include primary tumors from women between 40 and 70 years of age. We excluded samples with low tumor cell percentage (defined as < 50%) or a high level of necrosis (defined as > 5 %), ....."

Can the method be applied in more complex case? What is necessary to enlarge its application ? Discuss the limit of Gtex if any.

3/ On the other hand, the question in clinic practice is more often to perform a differential analysis between tumor sub-types to improve their classifications and to evaluate disease risk. The comparison with normal tissues is not a challenging diagnosis purpose.

Can the method be used in complex situation to refine the classification ? Can the authors comment this point ? Can they propose some used-case for their application in clinical practice ?

See methods p12: "As our model testing and comparison with DEseq2 pertained to the expression profile of BC subtype tissue vs. normal tissue (i.e., not between subtypes), we filtered the PAM50 dataset to only include the genes which were specific to a single subtype or which could distinguish BC subtype(s) from normal tissue."

Minor comments

1/Results part page 5 line 25

Can the authors explained why they use such metrics : "the representation space (or "latent space") has a dimensionality of 50, and representations are distributed according to a mixture of Gaussians with 45 components.

2/results part page 6 line 59

Do the author more clearly define what they called "positive control" experiment. in the sentence : To compare the sensitivities of DGD and DEseq2 in a "positive control" experiment

**Authors Response**

**Point-by-point responses to the reviewers’ comments:**

Reviewer 1:

The analysis of differential gene expression using bulk RNA sequencing data is important for diagnosing, predicting outcomes, and understanding diseases. But, often suitable control samples are missing. In the current manuscript, researchers propose a deep generative decoder model (DGD) based on [Schuster&Krogh, 2021+2022] that can substitute control samples by a so called generated "in silico closest normal sample". The model is trained on RNA-seq data from healthy tissues and learns a latent representation that effectively groups tissues together. The conclusions of the paper are adequately supported by the data shown: the paper demonstrates how the proposed approach successfully identifies cancer driver genes and marker genes specific to different subtypes of breast cancer with a high degree of specificity. The method outperforms a current state-of-the-art method DESeq2 in detecting differentially expressed genes. Genes identified using their model show significant enrichment for cancer-specific driver genes for most types of cancer in TCGA.

Interestingly, the in silico closest normal sample performs better than actual "normal" samples from the same cancer patients in TCGA data. Thus, the approach is a good alternative for DESeq2-based differential expression analysis, in particular for the quite relevant case that no normal control samples are available, i.e. for cancer samples of patients. I think the claims are somewhat far reaching, but as claimed in the paper quite well supported by a number of assessments and in-silico experiments. These experiments are clearly described in the paper and yield supportive results.

Sufficient details of the methods and evaluation are nicely described. It appears that the results could be reproduced. But, the actual reproduction of results is difficult. Code and models are available via a github repository mentioned in the manuscript. The documentation and application of the model could be made much easier and be much better documented in order to apply and reproduce the results/figures of the paper. The DGD model and its training requires more scripts and data described in previous papers of the authors. I am confident, that the model is trained and performs as described in the paper, but it is difficult to reproduce it from the tools provided. In order to be a useful alternative to standard applications of DESeq2 a better tutorial or scripts to apply the DGD model for the differential analysis of new samples is required.

*Response: We thank the reviewer for raising this. We agree that our model could be made more accessible and contain better documentation, which would improve the usefulness of the model and reproducibly. We have completely redesigned the Github repository and package in order to make our model easy to use across the field of transcriptomics. The new repository includes a command-line interface that makes the model easy to use and contains a tutorial in order to understand the software better. Finally, we have added the following to the Code Availability section of the manuscript:*

*“The source code is released under the GNU license and it is freely available at https://github.com/Center-for-Health-Data-Science/BulkDGD . The repository contains the source code,a command-line utilities and tutorials to facilitate its usage. Furthermore, we provide easy to install guidelines through the Python Package Index and conda.”*

The method is likely of broad utility: as mentioned above, the method is somewhat superior to the very widely used (in fact, the de-facto standard tool) DESeq2 applied with control samples. The method has better performance and can be applied when no controls are available or when control samples have not been measured.

The paper is of interest to a broad audience of biologists: The proposed method is a betteralternative to DESeq2, a standard tool typically applied for differential gene expression analysis (the DESeq2 paper published in Genome Biology is cited more than 50.000 times).

But it should be made much clearer how a broad audience could make easy use of the DGD model for standard DE analyses.

Scripts should be provided to reproduce the model, the data and/or the figures. The documentation of provided code and data should be improved in order to fulfill the claim of "a high potential for application" to (other/rare) diseases the application of deriving and using the DGD models should be much better supported by respective documentation, scripts and use cases.

*Response: We again agree with the reviewer that in order to support our “high potential for application”-claims the documentation and usability should be substantially improved. Please refer to the response above that we hope is sufficient.*

Reviewer 2:

The authors propose a novel approach to circumvent the problems and limitations of differential analysis of expression data. To date, the published methods lack reproducibility, making them more difficult to interpret and use in clinical practice. One of the difficulties in the gene differential analysis is the problem of the controls. The authors present a deep generative model (DGD) based on a previously developped method deposited in ArXiv (Schuster and Krogh 2022). The Deep Generative Decoder (DGD) is "a simple generative model that computes model parameters and representations directly via maximum a posteriori (MAP) estimation", when trained with bulk RNAseq data it returns a small-sized representation. Here, the novelty is to propose an application of the DGD model in the analysis of tumor RNAseq data and the possibility of the "gene expression model of normal tissue to replace control samples and to enable differential expression" . The authors' proposal is interesting for several aspects, strengthening the methods of analysis of RNAseq to facilitate its use in the clinic and offering the possibility of using the numerous public data to enrich and improve analyzes

However some questions need to be discussed, they mainly concern the limit of the approach.

1/ Can the author discuss the limit of the method starting from annotated genes or "raw gene count" from a reference. Rnaseq is a technology with a high potential to identify a great transcript diversity not fully accounted for even by the best reference databases. Each RNA is the result of a complex chain of events that combines by itself genetic variation, transcription regulation and RNA processing. To perform their analysis, the authors used the "raw gene count expression data from the Genotype-Tissue Expression (GTEx) and The Cancer Genome Atlas (TCGA) (downloaded from the Recount3 database (https://rna.recount.bio/ ( methods p11). Can the author precise the reference and comment its limit?

*Response: We thank the reviewer for the constructive comments. It is true that RNA-seq can give much more information about transcription than counts for annotated genes. To limit the output dimension of the model, we chose to use gene counts as given in the Recount3 database and further filtered the genes on expression etc as detailed in the paper. This is perhaps not so clear in the manuscript, and in the second line of “Results” we have added the sentence: “To limit the dimensionality of the model, we decided to use RNA-seq gene-level counts as defined in Recount3 [22] and further reduce the genes to a set of about 17000 (see Methods).”*

*Limiting the analysis to counts for a pre-specified subset of annotated genes of course limits the applicability to questions involving those genes, and the model cannot be used for other types of analyses. We added a paragraph at the end of the Discussion mentioning the limitations.*

2/ The second concern the GTEX cohort and the representation of cell subtypes. GTEx is an interesting dataset containing samples from 31 different human tissues. However each tissue, often originating from an organ in the Gtex cohort, is by itself a complex representation of a mixture of cell populations. A tumor type is a complex representation of the tissue and as such a complex mixture of cell types. Breast cancer is a good illustration of this diversity with Basal, Luminal A, Luminal B and Her2-enriched subtypes. In methods p12, the authors indicate : "To obtain a homogenous breast cancer (BC) dataset for testing, we curated the TCGA BC samples to only include primary tumors from women between 40 and 70 years of age. We excluded samples with low tumor cell percentage (defined as < 50%) or a high level of necrosis (defined as > 5 %), ....." Can the method be applied in more complex case? What is necessary to enlarge its application ? Discuss the limit of Gtex if any.

*Response: This is a very significant point, and we believe this is the most important reason that differential analysis is so difficult in bulk samples. As the reviewer is indicating, when samples with different proportions of cell types are compared, one will see a strong differential expression of genes that differ between the cell types – and often stronger than the difference between case and control. We believe the success of our model is that it learns this variation in normal samples, because the GTEx data contains large variability in these proportions. So when we find the closest in-silico normal sample for a cancer sample, it corresponds to the correct mixture of normal cells, and therefore it is possible to more clearly see how it differs from normal.*

*The statement that the reviewer is pointing to “To obtain a homogenous breast cancer (BC) dataset…” is probably a bit misleading, because the 381 samples are still quite heterogeneous in terms of cell mixture, etc. But we agree with the reviewer that our filter might affect the applicability of our method in clinical settings. We have extended our analysis to the complete TCGA breast cancer set (i.e. the same analysis without applying any filter), finding that our results are consistent with more complex and realistic scenarios. Interestingly, we also see that tumor purity is strongly associated with enrichment. For instance we see that the only samples with no enrichment are samples with 0% tumor purity, which in our minds further strengthen our results.*

*These results are added in a new supplementary figure (Supplementary Figure S3) and we added the following to the Results section:*

*“To ensure that the results were not due to a too restrictive filtering, we performed the same analysis without any filtering in the whole TCGA breast cancer dataset (Fig. S3). We found that our filtering scheme did not affect the enrichment scores (Fig. S3A). We also explored whether some of our filters (age, tumor purity, necrosis etc.) had any impact on the enrichment scores, finding, unsurprisingly, that a higher tumor purity is associated with a higher enrichment score in marker genes (Fig. S3B).”*

3/ On the other hand, the question in clinic practice is more often to perform a differential analysis between tumor sub-types to improve their classifications and to evaluate disease risk. The comparison with normal tissues is not a challenging diagnosis purpose. Can the method be used in complex situation to refine the classification ? Can the authors comment this point ? Can they propose some used-case for their application in clinical practice ? See methods p12: "As our model testing and comparison with DEseq2 pertained to the expression profile of BC subtype tissue vs. normal tissue (i.e., not between subtypes), we filtered the PAM50 dataset to only include the genes which were specific to a single subtype or which could distinguish BC subtype(s) from normal tissue."

*Response: In clinical practice, the tumors are subtyped from the differentially expressed genes (i.e. those that differ from normal). We hope that the improved accuracy in differential genes relative to normal can improve on the subtype classification using the existing marker genes. It is a very interesting question, whether the model can help to refine the classification, but we have not looked into that. However, we add a sentence in the discussion section to address the issue and clarify it:*

*“More importantly, we expect our model to improve the classification of different cancer subtypes. Subtype classification is performed based on which genes are differentially expressed and the ability to better detect differential expression should improve classification.”*

Can the authors explained why they use such metrics : "the representation space (or "latent space") has a dimensionality of 50, and representations are distributed according to a mixture of Gaussians with 45 components.

*Response: We are sorry that we were not very clear about this point. The dimension, number of mixture components, etc were chosen through the hyperparameter optimization described in Methods. We have rephrased the first paragraph of the Results section to hopefully make it more clear.*

Do the author more clearly define what they called "positive control" experiment. in the sentence : To compare the sensitivities of DGD and DEseq2 in a "positive control" experiment

*Response: We are sorry that this is not clear from the text. In this experiment, we are using the subtype annotation in TCGA as the ground truth and testing whether the genes associated with these subtypes behave as expected; it is known beforehand which genes should be up or down-regulated. This is why we call it a positive control, because we can test if the model predictions are consistent with this. We have expanded the explanation a bit in the paper and hope it is more clear.*

**Second round of review**

**Reviewer 1**

The authors have addressed the main points of the reviewers. @reviewer1: The code availability appears to be much improved with a new command-line interface, scripts and tutorials. Thereby, the main claim of the paper to provide an alternative to the widely used state-of-the-art method DeSeq2 is much better supported and, thus, hopefully the new approach and method will be of use for many differential analyses of high-through put data.

@reviewer2: Also the main points of this reviewer (in particular wrt to limitations of the method) have been addressed, although a bit sloppy and acknowledging that there are some limitations in particular for applications in clinical (e.g. for cancer subtyping) settings.

**Reviewer 2**

The authors respond to all the comments and include their corrections in the manuscrit. They also proposed additional results in a supplemental results (in figure S3) to respond to some questions.

For the list of annotated gene, can the authors indicate the precise reference and give the direct Github link ?

see "We further reduced the set of genes to protein-coding genes according to the the UCSC Genome Browser [44]. The gene list is available in our Github repository (see below). After preprocessing, the GTEx dataset contained 18,975 samples and 16,883 annotated protein-coding genes.? "

**Authors Response**

**Point-by-point responses to the reviewers’ comments:**

Reviewer 1:

The authors have addressed the main points of the reviewers. @reviewer1: The code availability appears to be much improved with a new command-line interface, scripts and tutorials. Thereby, the main claim of the paper to provide an alternative to the widely used state-of-the-art method DeSeq2 is much better supported and, thus,hopefully the new approach and method will be of use for many

differential analyses of high-through put data. @reviewer2: Also the main points of this reviewer (in particular wrt to limitations of the method) have been addressed, although a bit sloppy and acknowledging that there are some limitations in particular for applications in clinical (e.g. for cancer subtyping) settings.

*Response: We thank reviewer 1 for revising our manuscript and the nice comments.*

Reviewer 2:

The authors respond to all the comments and include their corrections in the manuscrit. They also proposed additional results in a supplemental results (in figure S3) to respond to some questions. For the list of annotated gene, can the authors indicate the precise reference and give the direct Github link ? see "We further reduced the set of genes to protein-coding genes according to the the UCSC Genome Browser [44]. The gene list is available in our Github repository (see below). After preprocessing, the GTEx dataset contained 18,975 samples and 16,883 annotated protein-coding genes.? "

*Response: We thank reviewer 2 for revising again our manuscript and for pointing out the availability of the gene list. We have now added added a link to the github repository.*
